# Maternal *n*-3 Polyunsaturated Fatty Acid During Pregnancy and Lactation: Linking Brain Lipid Biology to Cognitive Outcomes

**DOI:** 10.3390/nu18193142

**Published:** 2026-09-24

**Authors:** Yasna Muñoz Carvajal, Nicolás Palacios-Avendaño, Daniela Álvarez, Macarena Ortiz, Juan F. Montiel, Rodrigo Valenzuela, Manuel Maliqueo

**Affiliations:** 1 Laboratory of Endocrinology and Metabolism, Department of Internal Medicine West Division, Faculty of Medicine, University of Chile, Santiago 8350499, Chile; 2 Department of Nutrition, Faculty of Medicine, University of Chile, Santiago 8350499, Chile; 3 Laboratory of Neuroplasticity and Neurogenetics, Department of Biochemistry and Molecular Biology, Faculty of Chemical and Pharmaceutical Sciences, University of Chile, Santiago 8350499, Chile; 4 Laboratorio de Neurociencias Integradas, Centro de Investigación Biomédica, Facultad de Medicina, Universidad Diego Portales, Santiago 8370007, Chile

**Keywords:** docosahexaenoic acid, *n*-3 polyunsaturated fatty acids, pregnancy, lactation, placental transport, brain development, cognitive development, MFSD2A

## Abstract

Adequate maternal nutrition during pregnancy and lactation is essential for fetal and infant brain development. Among dietary lipids, *n*-3 polyunsaturated fatty acids (*n*-3 PUFAs), particularly docosahexaenoic acid (C22:6*n*-3, DHA), are of major biological relevance because this FA is preferentially transferred from the mother, accumulates rapidly in the developing brain, and contributes to membrane organization, neuronal differentiation, synaptic maturation, and lipid-mediated signaling. This review integrates current evidence on maternal *n*-3 PUFA metabolism during pregnancy and lactation, placental and blood–brain barrier transport of DHA, and the mechanisms linking cerebral *n*-3 PUFA availability to neurodevelopment and cognitive function. Attention is given to selective DHA transport, brain lipid remodeling, synaptic plasticity, neuroimmune regulation, and the translation of experimental findings into human developmental outcomes. Across experimental and human studies, the clearest functional signal emerges under developmental *n*-3 PUFA deficiency or defined biological challenge, whereas supplementation above nutritional adequacy produces smaller and less consistent cognitive effects. These findings support a context-dependent model in which the functional consequences of *n*-3 PUFA availability are shaped by baseline nutritional status, developmental timing, brain region, biological challenge, and the assessed cognitive domain. Accordingly, DHA is best understood as an essential component of normal brain development rather than as a nutrient whose supplementation necessarily enhances cognition above nutritional adequacy.

## 1. Introduction

Pregnancy and lactation represent critical developmental windows during which maternal nutrition contributes to fetal growth and to the structural and functional maturation of the developing nervous system [1,2,3]. During these periods, the brain undergoes rapid cellular proliferation, differentiation, synaptogenesis, and circuit maturation, increasing its dependence on an adequate, continuous supply of nutrients [4,5]. Nutritional disturbances during these sensitive stages may therefore influence neurodevelopmental trajectories and contribute to persistent changes in cognitive, motor, and socioemotional function [6]. Accordingly, maternal nutritional status and dietary quality constitute important determinants of the intrauterine and early postnatal environments in which brain development occurs.

Among the nutrients required during these developmental windows, lipids are particularly relevant because they provide not only metabolic substrates but also structural and signaling components required for fetal organogenesis and brain development [7,8,9]. Through gestation, maternal lipid metabolism undergoes profound physiological adaptations. The first trimester is characterized by an anabolic phase marked by enhanced lipid synthesis and tissue storage, whereas the second and third trimesters transition into a catabolic state to mobilize energy substrates in preparation for parturition [10]. Consequently, lipids serve not only as vital caloric reserves to meet maternal–fetal metabolic demands but also as essential structural and functional components for fetal organogenesis [10]. Among these, *n*-3 PUFAs are particularly relevant because the essential precursor α-linolenic acid (C18:3 *n*-3, ALA) can be converted to longer-chain derivatives, including docosahexaenoic acid (C22:6*n*-3, DHA), although endogenous conversion is limited and DHA availability therefore depends substantially on dietary supply [11,12,13,14,15]. As one of the principal long-chain PUFAs (20–22 carbon atoms) in the central nervous system, DHA is indispensable for brain architecture, neurophysiological function, cognitive protection, and the regulation of inflammatory pathways. Accordingly, perturbations in cerebral DHA accretion or status may precipitate neurological disorders or functional deficits [16,17]. Despite the well-established biological relevance of DHA during neurodevelopment, important questions remain regarding how maternal *n*-3 PUFA availability is translated into fetal and cerebral DHA status and, ultimately, into functional outcomes [18]. This relationship is shaped by maternal metabolism, placental transfer, postnatal supply, and selective brain uptake, and may be further modified by nutritional status and developmental context [17,19]. This process is mediated by a coordinated network of fatty acid (FA) transporters and binding proteins that facilitate the uptake, intracellular transport, and delivery of DHA from the maternal circulation to the fetus via the placenta and subsequently to the brain [20,21,22]. However, although experimental studies consistently support a biological requirement for *n*-3 PUFAs during neurodevelopment, the cognitive benefits of supplementation in humans are less consistent, highlighting a translational gap between biological requirement and measurable neurodevelopmental benefit.

Therefore, this review integrates evidence across the maternal–placental–brain axis, from *n*-3 PUFA metabolism and DHA transport during pregnancy and lactation to brain lipid organization, neural plasticity, and cognitive development, with particular emphasis on distinguishing established mechanisms from unresolved questions and reconciling experimental and human evidence. In Figure 1 summarizes the maternal-placental-cerebral axis that links the availability of *n*-3 polyunsaturated fatty acids to the transport of DHA during neurological development.

## 2. Methodology

A narrative literature review was conducted using PubMed, Scopus, Web of Science, and Ovid MEDLINE. Searches combined terms related to *n*-3 polyunsaturated fatty acids (*n*-3 PUFAs), DHA, pregnancy, lactation, placental transport, blood–brain barrier transport, MFSD2A/Mfsd2a, brain development, neuroinflammation, synaptic plasticity, and cognitive outcomes. Recent literature was prioritized to reflect the current state of the field, while seminal studies were included when necessary to establish fundamental mechanisms of *n*-3 PUFA metabolism, placental and brain transport, membrane biology, or neurodevelopment. Studies were selected according to their relevance to the maternal–placental–brain axis and their contribution to mechanistic, developmental, or translational aspects of *n*-3 PUFA biology. Evidence from human observational studies and randomized controlled trials was integrated with experimental evidence from animal models and cellular systems. Evidence was synthesized narratively by developmental stage, biological level, and study design, with particular attention to convergent findings, discrepancies between experimental and human evidence, and factors that may contribute to heterogeneous outcomes. Duplicate publications, conference abstracts, study protocols, and overlapping reports of the same study population and outcomes that did not provide additional relevant information were excluded. Because this work was designed as a narrative rather than a systematic review, no formal risk-of-bias assessment was performed; however, studies were critically evaluated with respect to study design, experimental model or population, characterization of *n*-3 PUFA exposure or status, developmental timing, and the relevance of the reported outcomes. The literature search was updated through July 2026.

## 3. Metabolism of *n*-3 Polyunsaturated Fatty Acids During Pregnancy and Lactation and Their Relationship to the Brain

Maternal lipid metabolism during an uncomplicated pregnancy is divided into phases. The anabolic phase occurs during the first two trimesters of pregnancy and is associated with increased maternal storage of FAs, triglycerides, phospholipids (PLs), cholesterol, and lipoproteins to ensure a continuous supply of nutrients to the growing fetus. During this phase, increased levels of estrogen, progesterone, and insulin in the maternal circulation promote lipogenesis and inhibit lipolysis, leading to a significant increase in plasma lipid concentrations during pregnancy [23]. The catabolic phase begins during the third trimester and involves an increase in lipolysis and a decrease in lipoprotein lipase (LPL) activity in adipose tissue, which increases the release of non-esterified free FAs, PL, and cholesterol into the maternal circulation to be transported to the fetus [24]. The concentration of DHA in the plasma PL and erythrocytes of the mother begins to increase very early in pregnancy, regardless of the diet. This increase results from an early change in maternal metabolism to meet the needs of rapidly proliferating cells, particularly those of the fetal brain [25]. The metabolic requirements for *n*-3 PUFAs increase by 10% during pregnancy and lactation, respectively [25]. According to the joint recommendations of the World Association of Perinatal Medicine, the Early Nutrition Academy, and the Child Health Foundation, maternal DHA consumption during pregnancy should be at least 200 mg/day. Nutritional supplements are among the suggested sources for achieving this goal [26].

FAs provide the fetus with energy, a substrate for the formation of plasma membranes, and eicosanoid precursors [27]. Maternal–fetal transport of FAs, which circulate in the form of unesterified FAs bound to albumin, can cause them to dissociate from albumin and cross the plasma membrane of the syncytiotrophoblast. However, most FAs are esterified to triglycerides and phospholipids [28]. These FAs are hydrolyzed by lipases for their release from maternal circulating lipoproteins, LPL and endothelial lipase (LE), hydrolyzing triglycerides and phospholipids, respectively [29]. Different proteins found in the trophoblast plasma membrane, such as FA translocase (FAT/CD36), FA-binding protein (FABPpm), and FA transporter protein (FATP), have been described as transporters of FA, while FABP is recognized as an intracellular binding protein [30]. Because DHA accumulates rapidly in the developing nervous system, adequate maternal–fetal supply is particularly important during late gestation and early postnatal life [31,32]. DHA is synthesized from ALA through a series of reactions described above; in the fetus, this enzymatic pathway is insufficient to provide sufficient DHA until 16 weeks after birth. Therefore, the concentration of DHA in the fetus depends on the amount of this FA consumed by the mother [33]. Also, these enzymes (D5- and D6-destaurases) are expressed at very low levels in placental tissue [34], so they are likely supplied via maternal–fetal placental transfer [35]. The period of DHA accumulation in humans occurs primarily during the third trimester of pregnancy and up to the first 6 to 10 months after birth; this is explained by increased mobilization of the fatty acid from maternal adipose tissue [31,36]. In vivo studies on maternal–fetal transfer of FA, conducted by administering FA labeled with stable isotopes, demonstrate a preferential transfer of DHA over other FAs, such as palmitic acid (C16:0), oleic acid (C18:1*n*-9, OA), and linoleic acid (C18:2*n*-6, LA); this may be due to increased levels of lysophospholipids in maternal plasma that are enriched in PUFAs [37,38]. This selective enrichment of PUFA in the fetal circulation is known as biomagnification [36]. It has been proposed that there exists an increase in the activity of the enzymes involved in the synthesis of DHA, together with an enhanced mobilization of DHA from maternal adipose tissue. Leptin from placental tissue also regulates the mobilization of FAs—primarily DHA—from adipose tissue in the fetus [39].

Breast milk composition is influenced by maternal factors, such as age, weight, and diet, and it changes throughout the lactation period [40]. As in pregnancy, determining maternal fatty acid balance during lactation is due to the ability of the mother to provide *n*-3 PUFAs from body stores and endogenous synthesis [41]. Recent evidence suggests that the *n*-3 PUFA composition of human milk cannot be explained solely by recent maternal dietary intake, supporting a contribution from maternal adipose tissue stores [42]. In addition, maternal DHA status is associated with DHA availability in human milk, emphasizing the importance of maternal DHA status in determining the supply of DHA to the breastfed infant [43]. Whereas colostrum is characterized by a high content of *n*-6 and *n*-3 PUFAs, specifically LA and ALA, mature human milk shows that the main PUFAs are DHA and *n*-3 docosapentaenoic acid (C22:5*n*-3, DPA) from the *n*-3 series [23,44,45]. However, *n*-3 PUFAs, including DHA, decreased from the 4th month of lactation [23]. In Figure 2 summarizes maternal lipid metabolism and the supply of DHA by the placenta during pregnancy.

### 3.1. DHA Supply During Early Brain Development

DHA is highly enriched in neuronal membranes and accumulates rapidly in the developing brain, particularly during late gestation and early postnatal life [46,47]. Because endogenous synthesis from ALA is limited during these developmental stages, fetal and neonatal brain DHA status depends substantially on maternal availability, placental transfer, and postnatal dietary supply [48,49,50]. Consequently, adequate cerebral DHA accretion requires not only sufficient maternal and infant intake but also efficient transfer across the placenta and subsequent uptake across the blood–brain barrier. These transport mechanisms are discussed in the following section.

DHA is a component of neuronal membranes and the retina [46,47]. It is estimated that the brain accumulates 905 mg of DHA from birth, so it is estimated that during the postnatal period, 5 mg/day should be incorporated, and an intake of 100 mg/day of DHA is recommended during the first year of life for the correct brain development of the child [48]. The biological activity of *n*-3 PUFAs has been observed in pregnancy and lactation, improving their bioavailability to the fetus, positively influencing infant neurodevelopment, because the developing brain can capture, desaturate and elongate linolenic acid to DHA to be incorporated into membrane phospholipids; also the efficiency of DHA from the diet is higher at this stage and this is added to a higher activity of the enzyme Δ6 desaturase during the perinatal period [49,50]. DHA availability supports the structural and functional maturation of the developing brain, as discussed in greater detail in Section 5.

As mentioned above, endogenous fetal synthesis is insufficient to meet developmental DHA requirements, so DHA is obtained from maternal intake and, during the postnatal period, is transferred through breast milk [51]. Barrera et al. evaluated the levels of FAs in breast milk and their intake in 50 pregnant women during the 6th month of pregnancy until the 6th postpartum month, and observed that pregnant women had a high intake of *n*-6 PUFAs (LA and arachidonic acid (C20:4*n*-6, ARA)) and a low intake of *n*-3 with an unbalanced *n*-3:*n*-6 ratio, which generates a decrease in the levels of DHA in erythrocytes and breast milk [8]. In addition, Chamorro et al. demonstrated that maternal obesity reduced the DHA and ARA content in breast milk and maternal erythrocytes [52]. Markhus et al. conducted a study to evaluate the changes in FAs from pregnancy to 12 months postpartum associated with seafood consumption and observed a variation in DHA and ARA levels during the time of evaluation, with seafood consumption being lower than the recommendation, thus inferring that from 6 months postpartum, DHA levels in the mother are replenished [53]. Additionally, the intake of *n*-3 PUFAs by the mother has positive effects for the offspring, evidence for which is demonstrated in the studies of Hussain et al. and Sardinha et al., who evaluated the effect of a maternal diet rich in *n*-3 PUFAs in an animal model from pregnancy to weaning in relation to body composition, observing reductions in the body fat of the offspring with reductions in the levels of cholesterol, triglycerides and insulin resistance [54,55]. Double-blind controlled clinical trials have also evaluated the positive effect of supplementation with DHA plus eicosapentaenoic acid (C20:5*n*-3, EPA) administered through fish oil capsules containing 2 g of *n*-3 in pregnant women during the second trimester until delivery, showing longer gestational duration, higher birth weight, and higher fat-free mass in newborns assessed 3 days after birth, with higher *n*-3 PUFA levels associated with lower adiposity in offspring [56,57]. Schaefer et al. evaluated the DHA, lutein, and micronutrient content of breast milk from 70 lactating women over 12 weeks of supplementation and observed significant increases in DHA levels in the milk and maternal blood [58]. Likewise, dietary education on the importance of DHA for new mothers increased the content of this FA in breast milk [59]. A positive effect of *n*-3 PUFAs on brain development has been evidenced. A study evaluated 52 mothers and their 3-month-old infants in relation to the PUFA content of human milk and found that infants whose mothers had high levels of *n*-3 PUFAs (specially DHA) in breast milk showed low levels of negative affect, which is associated with a lower risk of internalizing disorders (with high levels of negative affectivity, including depressive disorders, anxiety disorders, among others) in early childhood [60]. In summary, *n*-3 PUFAs provide benefits during pregnancy and lactation, thanks to their biological mechanisms, such as neuronal membrane fluidity and synaptic modulation, among which their anti-inflammatory properties stand out through the inhibition of the NF-κB transcription factor, which leads to a reduction in pro-inflammatory markers and prevents the development of diseases such as obesity, insulin resistance, and cardiovascular diseases [51]. The scientific evidence shows that *n*-3 FAs, especially DHA, are essential nutrients during reproduction, pregnancy, and lactation, due to their critical role in brain development, neural plasticity, and the metabolic programming of offspring. Since fetal and neonatal intake largely depends on maternal nutrition, ensuring adequate intake of these FAs is a key strategy for promoting maternal and child health and supporting optimal development in the early stages of life.

### 3.2. n-3 Polyunsaturated Fatty Acid Transport by MFSD2A in Placenta and Brain

Following maternal supply and placental transfer, DHA must reach the developing brain through selective transport mechanisms. MFSD2A is a sodium-dependent lysophospholipid transporter required for efficient DHA uptake across the blood–brain barrier [21]. In the placenta, MFSD2A also functions as a receptor for syncytin-2, suggesting additional roles in trophoblast fusion and placental development [61]. Emerging evidence further indicates that placental MFSD2A expression is associated with fetal DHA availability. In women with treated gestational diabetes, lower placental MFSD2A expression has been associated with reduced cord-blood DHA concentrations [62]. Maternal obesity may also alter placental DHA handling, although available findings are not fully consistent and appear to vary according to fetal sex and experimental model [63,64,65,66,67,68,69].

This is primarily due to hyperlipidemia, which affects lipid metabolism. However, a study evaluating FA uptake in placental explants shows that radiolabeled DHA uptake is unaffected by maternal obesity or fetal sex [68]. On the other hand, a preliminary metabolomic study of the placenta in obese women showed increased PA concentrations and decreased DHA, ARA, and stearic acid [69]. A specific placental phenotype is observed in obese women, which warrants further research.

At the brain level, lipid uptake across the blood–brain barrier (BBB) is essential for the accumulation of DHA in the brain. In recent times, it has been proposed that the major uptake of this FA occurs through MFSD2A [70], which is expressed in a large number of tissues and which captures lysophospholipids through the BBB, by a group of high-density endothelial cells that compose the cerebral microvasculature, and which regulates the entry of macromolecules from the blood to the brain [47,71,72]. As mentioned earlier, a high percentage of DHA is transported to the brain via this transporter, confirming the physiological role of MFSD2A [21]. In cases of obesity, a study in mice observed an increase in the Mfsd2a transporter under conditions of obesity in the hypothalamus and paraventricular nucleus, but not in DHA transport; therefore, it is inferred that there is some compensatory mechanism by which the brain can protect itself from the effects of obesity [73]. One possible explanation for this phenomenon is that DHA regulates its own synthesis by inhibiting the enzyme elongase; this may shed light on what happens when there is a deficiency of DHA in the diet [74]. Table 1 shows the main differences between normal pregnancy and pregnancy in women with obesity.

## 4. From Brain Lipid Architecture to Neural Function: Roles of *n*-3 and *n*-6 PUFAs in the Cortex and Hippocampus

### 4.1. Selective Brain Incorporation and Membrane Organization

DHA and ARA are the predominant long-chain polyunsaturated fatty acids (LC-PUFAs) in brain membranes, where their esterification into specific phospholipid classes supports membrane organization, signaling, and neurotransmission [76]. Their distribution is both molecularly and regionally selective: DHA and ARA are abundant in ethanolamine phospholipids, DHA is relatively enriched in phosphatidylserine, and ARA is prominent in phosphatidylinositol and phosphatidylcholine; compared with the cortex, the hippocampus is enriched in several abundant ARA-containing species but relatively depleted in many DHA-containing phospholipids [77]. This organization is maintained through distinct deacylation–reacylation pathways, as ARA is preferentially incorporated into phosphatidylinositol and mobilized mainly through Ca^2+^-dependent cytosolic phospholipase A_2_ (cPLA_2_), whereas DHA is enriched at the sn-2 position of phosphatidylethanolamine, phosphatidylserine, and plasmalogens and released predominantly through Ca^2+^-independent phospholipase A_2_ (iPLA_2_) pathways, allowing both pools to respond to common neuronal or glial stimuli while remaining metabolically distinct [78,79,80,81]. Beyond regulating lipid signaling, the physiological pairing of a saturated chain at sn-1 with a PUFA at sn-2 preserves membrane integrity while enabling curvature and vesiculation, with DHA-containing phospholipids producing greater deformability than ARA-, ALA- or LA-containing species [82].

In contrast, EPA remains scarce in brain phospholipids despite entering the brain at rates comparable to those of DHA, because it is rapidly de-esterified, poorly recycled, and redirected toward β-oxidation or elongation to *n*-3 DPA [83,84,85]. Thus, EPA appears to contribute less as a stable structural component than through transient availability or downstream metabolites, although this functional interpretation remains less directly established than its rapid metabolic turnover [84,85]. Likewise, although ALA and LA are precursors of DHA and ARA, respectively, their conversion within the brain is limited, and their effects on cerebral LC-PUFA composition depend strongly on peripheral metabolism and brain-specific homeostatic mechanisms [86,87]. Brain DHA is therefore relatively resistant to moderate changes in precursor intake, whereas severe ALA restriction reduces circulating DHA availability, net brain DHA incorporation, and phospholipid DHA concentrations while promoting compensatory accumulation of n-6 docosapentaenoic acid (C22:5*n*-6, *n*-6 DPA) [87]. Collectively, these findings indicate that brain PUFA composition is not merely structural but is actively maintained through selective incorporation, remodeling, and metabolic retention, thereby shaping the membrane environment in which neuronal differentiation, synaptic assembly, and activity-dependent plasticity occur.

### 4.2. Neuronal Development and Synaptic Plasticity

Building on their selective incorporation into neuronal membranes, DHA and ARA influence partially distinct stages of neuronal and synaptic maturation. In embryonic hippocampal cultures, low-micromolar DHA increased neurite length and branching, whereas ARA, *n*-6 DPA, and OA did not reproduce this early morphogenic effect; moreover, neurite outgrowth occurred below the concentrations required for retinoid X receptor α (RXRα) activation, suggesting that direct RXRα activation is unlikely to be the primary mechanism, although involvement of other RXR isoforms or DHA-derived metabolites cannot be excluded [88,89]. DHA supplementation further increased synapsin-positive puncta, synaptic protein expression, and glutamatergic spontaneous activity, whereas developmental *n*-3 depletion reduced hippocampal synapsins and glutamate-receptor subunits and impaired long-term potentiation [90]. In contrast, ARA promoted dendritic filopodia and spine formation, synaptic marker expression, and actin-regulatory proteins in cultured hippocampal neurons, and independently induced a long-lasting, long-term potentiation-like (LTP-like) facilitation of CA1 transmission through PKC-dependent nicotinic receptor signaling and increased presynaptic glutamate release [91,92]. Together, these findings suggest that DHA and ARA are not interchangeable trophic substrates, but may support complementary stages of early neurite development, synaptic assembly, and functional maturation.

These effects are also regionally and mechanistically selective rather than reflecting a generalized enhancement of synaptic efficacy. Lifelong *n*-3 PUFA deficiency abolished endocannabinoid-mediated long-term depression in the prelimbic prefrontal cortex through functional uncoupling of presynaptic cannabinoid receptor type 1 (or CB1 receptors), while sparing basal glutamatergic transmission, AMPA/NMDA balance, mGluR2/3-mediated depression and CB1 receptor abundance; importantly, the same receptor defect was not detected in the motor cortex [93]. Dietary *n*-3 PUFA restriction altered neurotrophin and kinase signaling in a region-dependent manner across the frontal cortex and hippocampus [94,95], whereas maternal fish-oil supplementation under high-fat-diet exposure attenuated cortical spine alterations in a sex- and region-dependent manner [96]. Together, these findings indicate that PUFA-dependent plasticity is shaped by developmental stage, brain region and synaptic context, rather than reflecting a uniform response to altered *n*-3 PUFA availability.

### 4.3. Lipid Signaling, Neuroimmune Regulation, and Neuronal Homeostasis

PUFAs released from neural membrane phospholipids generate bioactive mediators that regulate inflammatory amplification, resolution, and neural survival. ARA serves as a precursor for prostaglandins, leukotrienes, and thromboxanes, but also for lipoxins and endocannabinoids with pro-resolving or neuromodulatory functions, whereas DHA gives rise to specialized mediators including neuroprotectin D1, D-series resolvins, and the ethanolamide synaptamide [97,98]. Synaptamide suppresses NF-κB-dependent inflammatory responses through GPR110/ADGRF1–cAMP signaling, while studies in adult neuroinflammatory models show preservation of hippocampal neurogenesis, dendritic architecture, and synaptic plasticity [98,99,100,101]. Neuroprotectin D1 promotes pro-survival cREL–BIRC3 signaling and has also been linked to Bcl-2-family pathways, whereas resolvin D1 attenuates inflammation-associated blood–brain barrier disruption through an FPR2-dependent pathway associated with A20 upregulation and suppression of NF-κB/NLRP3 signaling [102,103,104]. Thus, DHA-derived mediators constitute a functionally diverse network whose effects depend on receptor identity, cellular target, and type of neural challenge.

Developmental and sustained availability of *n*-3 PUFAs also shapes neuroendocrine and metabolic signaling across brain regions. Deficiency alters glucocorticoid-, vasopressin- and neurotrophin-related signaling, including GR, NPY1R, Avp, Avpr1b, TrkB–CREB and insulin pathways, with marked regional differences across the frontal cortex, hippocampus and hypothalamus [94,105]. In the prefrontal cortex, *n*-3 PUFA deficiency induces HPA-axis hyperactivity, reduces GR, BDNF, and FKBP51 expression, and causes selective apical dendritic atrophy, effects attenuated by lowering corticosterone or by *n*-3 PUFA supplementation during chronic stress [106]. Attenuation of cortical morphological alterations by maternal fish-oil (as source of *n*-3 PUFAs) supplementation under high-fat-diet exposure occurred without detectable changes in *Nfkb1* or *Il1b* transcripts, indicating that developmental protection by *n*-3 PUFAs need not coincide with canonical inflammatory readouts [96].

This distinction is particularly relevant during pregnancy, lactation and early postnatal life, when *n*-3 PUFA availability contributes to microglial and neuronal developmental programs. Perinatal *n*-3 PUFA deficiency alters hippocampal microglial homeostasis and can promote excessive complement- and 12/15-LOX/12-HETE-related synaptic engulfment, accompanied by reduced CA1 spine density and impaired spatial working memory; however, its effects are compartment-specific, as microglia–myelin interactions are instead reduced in developing white matter [107,108,109]. Maternal *n*-3 PUFA status also influences hippocampal cell proliferation, neuronal maturation, and survival, while early deficiency can leave persistent BDNF-related alterations despite later restoration of brain DHA and can increase vulnerability to prenatal inflammatory challenge [110,111,112,113].

Translation to humans remains less direct. Prenatal EPA+DHA supplementation can produce persistent changes in cerebral physiology without corresponding differences in cognition or psychopathology, whereas observational studies associate low *n*-3 PUFA status with inflammatory and structural brain abnormalities [114,115]. Collectively, these findings indicate that *n*-3 PUFA availability regulates cerebral resilience through coordinated effects on lipid mediators, microglial remodeling, neuroendocrine adaptation, metabolism and neuronal survival rather than through a simple opposition between beneficial *n*-3 and harmful *n*-6 PUFAs. Importantly, developmental evidence is substantially stronger for overall *n*-3 availability and brain DHA status than for EPA-specific actions, which remain comparatively unresolved. In Figure 3 shows a summary maternal DHA supply, brain uptake, and DHA-derived bioactive mediators during neurodevelopment.

## 5. Implications of *n*-3 PUFAs for Cognitive Development

### 5.1. Prenatal n-3 PUFA Exposure and Cognitive Development

Large randomized trials including DOMInO, POSGRAD and DHANI reported no improvement in broad infant developmental outcomes after maternal DHA supplementation, despite effective modification of maternal or offspring DHA exposure in several of these trials [116,117,118]. Longer follow-up and more domain-specific testing suggest that subtler effects may nevertheless emerge: in POSGRAD, prenatal DHA supplementation was associated with fewer omission errors on an objective sustained-attention task at 5 years, whereas global cognition and parent-reported behavior were unchanged and most attention-related outcomes did not remain significant after correction for multiple comparisons [119]. Observationally, higher first-trimester maternal *n*-3 PUFA intake in the INMA cohort was associated with modestly higher general cognition, verbal and executive-function scores at 4 years and better sustained-attention performance at 7 years, although maternal education and socioeconomic position remained important potential confounders and the exposure reflected total *n*-3 intake rather than DHA specifically [120]. Similarly, higher total maternal DHA exposure from diet and supplements has been associated with better cognitive performance at 12 months despite the absence of a clear effect of randomized supplementation itself, suggesting that total nutritional exposure may be relevant but also introducing potential confounding by maternal diet, supplement use, and home environment [121]. The main paradigms for prenatal human and animal interventions, exposure windows, and cognitive outcomes are summarized in Table 2.

**Table 2:** Developmental *n*-3 PUFA exposure paradigms and functional outcomes in animal and human studies.

Paradigm	Intervention/ Exposure	Model/Population	Exposure Window	Main Functional Finding	Reference
Supplementation	Maternal supplementation: 800 mg DHA + 100 mg EPA/day	Human	<21 GW → delivery	Global cognition and language =; cognitive delay ↓‡	[116]
Supplementation	Maternal supplementation: 400 mg algal DHA/day	Human	18–22 GW → delivery	18 mo: mental/psychomotor/behavioral development =; 5 y: global cognition/behavior =; K-CPT omission errors ↓	[118,119]
Supplementation /exposure-response	DHA 300 mg/d + EPA 67 mg/d; total DHA intake also analyzed	Human	24–28 GW → 3 mo lactation	Randomized supplement =; total DHA ≥ 600 mg/d → 12-mo cognition ↑†	[121]
Supplementation	400 mg algal DHA/day	Human	≤20 GW → 6 mo postpartum	DQ =; mental score =; motor score =	[117]
Supplementation	*n*-3 LC-PUFA (EPA, DHA)	Human	GW24 → 1 wk postpartum	No metabolic–cognitive/psychopathology associations	[115]
Observational exposure	Maternal dietary ALA + EPA + DHA intake	Human	1st and 3rd trimesters	1 y: BSID =; higher 1st-trimester *n*-3 intake → 4-y cognition/verbal/executive ↑ and 7-y attention variability ↓†	[120]
Supplementation	Control vs. DHA vs. DHA+ARA formula	Human	≤1 wk of age → 12 mo	Prior 14-mo assessment: DHA-only vocabulary ↓; at 39 mo: IQ, receptive/expressive language, visual-motor function, and visual acuity =	[122]
Deficiency	*n*-3-deficient vs. *n-*3-adequate diet	Rat	Gestation + lactation + postweaning	Brain DHA ↓ 58%; acquisition mildly impaired; retention =; reversal learning markedly impaired/perseveration ↑	[123]
Supplementation	Fish-oil *n*-3 supplementation (EPA, DHA)	Rat	Conception → PND21	Acquisition ↑; probe/reference memory =; DG PS-LTP ↑	[124]
Challenge/rescue	Perinatal *n*-3-enriched diet ± neonatal sevoflurane	Rat	*n*-3: GD2 → PND14; sevo: PND7	Sevo: working/short-term memory ↓; +*n*-3: attenuation; spatial/reference memory =	[125]
Deficiency/repletion	DEF/SUF crossover ± COX inhibition	Mice	F1–F3; F3 crossover at PND21 for 16 wk	DEF: spatial recognition ↓; DEF→SUF restored; SUF→DEF impaired; COX inhibition rescued	[126]
Deficiency/mechanistic	Maternal *n*-3-deficient vs. sufficient diet	Mice	Gestation + lactation → PND21	Spatial working memory ↓; CA1 spines ↓; excessive complement- and 12/15-LOX- related synaptic engulfment	[107]
Deficiency × challenge	*n*-3 deficiency ± maternal immune activation	Mice	Diet: gestation → PND21; LPS: E17	*n*-3 deficiency: learning ↓; DEF + MIA: spatial memory ↓ and hyperlocomotion ↑	[113]
Challenge/rescue	Maternal *n*-3 mixture (EPA-rich) ± Pb exposure	Mice	*n*-3: GD3 → ~PND13; Pb: GD5 → PND14	Pb: learning/spatial and working memory ↓; +*n*-3 attenuated deficits; *n*-3 alone ≈ control	[127]
Timing comparison	*n*-3; different onset windows	Rat	Preconception/GW1/GW2/GW3/lactation	Early acquisition differed among groups; later acquisition/probe memory =; no discrete critical window established	[128]

**Abbreviations:** α-linolenic acid (ALA); arachidonic acid (ARA); cornu ammonis 1 (CA1); cyclooxygenase (COX); deficient *n*-3 diet (DEF); dentate gyrus (DG); docosahexaenoic acid (DHA); developmental quotient (DQ); eicosapentaenoic acid (EPA); gestational day (GD); gestational week (GW); intelligence quotient (IQ); Conners’ Kiddie Continuous Performance Test (K-CPT); Bayley Scales of Infant Development (BSID); lipopolysaccharide (LPS); lipoxygenase (LOX); maternal immune activation (MIA); lead (Pb); population-spike long-term potentiation (PS-LTP); postnatal day (PND); sufficient *n*-3 diet (SUF); years (y); months (mo); weeks (wk). ↑, increased/higher; ↓, decreased/lower; =, no statistically significant difference; ≈, approximately comparable/no statistically significant difference; ±, with or without; →, progression, exposure interval, or dietary crossover, as applicable. Other K-CPT outcomes and clinical risk measures did not differ significantly after correction for multiple comparisons. † Association/exposure analysis rather than randomized causal effect. ‡ Secondary outcome.

Animal intervention studies are therefore particularly informative because they allow controlled manipulation of exposure timing and nutritional background while directly linking maternal *n*-3 PUFA availability to offspring brain function. In healthy offspring, maternal *n*-3 PUFA supplementation improved spatial-learning acquisition and dentate-gyrus LTP without uniformly enhancing memory retention, whereas under prenatal lead exposure or neonatal sevoflurane it produced larger benefits for spatial and working memory together with preservation of BDNF-related signaling, antioxidant defenses, neurogenesis and neuronal survival [124,125,127]. Manipulating the onset of supplementation across preconceptional and gestational stages also produced differences in early Morris water-maze acquisition, but the absence of effects on probe-trial reference memory and important baseline and litter-related limitations preclude identification of a discrete critical window [128]. These experimental findings provide causal support for domain- and context-dependent effects of developmental *n*-3 PUFA availability, a distinction considered further below.

### 5.2. Early Postnatal n-3 PUFA Exposure and Cognitive Development

Postnatal exposure provides an additional opportunity to influence brain *n*-3 and *n*-6 PUFA availability during a period of rapid synaptic and cortical maturation, but evidence from infant supplementation again indicates that increasing LC-PUFA exposure does not necessarily translate into broad cognitive enhancement. In term infants randomized to unsupplemented formula, DHA alone or DHA + ARA during the first year of life, IQ, receptive and expressive language, visual–motor performance and visual acuity did not differ at 39 months, and an earlier difference in vocabulary observed with DHA alone at 14 months was no longer detectable, suggesting that isolated early differences may be transient or test-dependent [122]. This lack of global benefit should not be interpreted as evidence that postnatal LC-PUFA supply is biologically irrelevant, because conversion of ALA and LA is insufficient to reproduce the DHA and ARA tissue status of breast-fed infants, and dietary DHA directly influences cerebral DHA accretion during infancy [129]. More focused cognitive paradigms have nevertheless identified effects on sustained attention and means-end problem solving, with outcomes varying with exposure duration and no consistent DHA dose–response relationship [129]. Thus, as observed for prenatal supplementation, postnatal *n*-3 PUFA exposure appears more likely to influence selected cognitive processes than global developmental performance, with outcomes strongly dependent on DHA–ARA composition, exposure duration, developmental stage, socioeconomic environment, and the sensitivity of the neuropsychological assessment. Postnatal intervention designs and their corresponding cognitive outcomes are also summarized in Table 2.

### 5.3. n-3 PUFA Deficiency, Nutritional Adequacy, and Translation to Human Outcomes: Reconciling Animal and Human Evidence

A clearer functional signal emerges when developmental *n*-3 availability is examined in terms of deficiency rather than supplementation above adequacy. Rats exposed to an *n*-3 PUFA-deficient diet from gestation through postnatal development showed a 58% reduction in brain DHA, modest impairment during initial spatial learning, and a substantially greater deficit in reversal learning, with increased errors and perseverative responses [123]. This domain specificity is consistent with intervention studies (reviewed in the previous sections) showing modest effects in otherwise healthy offspring but larger behavioral effects when *n*-3 PUFA supplementation is tested against defined developmental insults [124,125,127]. Importantly, these effects are not necessarily irreversible. In mice maintained for three generations on an *n*-3 PUFA-deficient diet, postweaning repletion for 16 weeks restored spatial recognition memory to levels comparable to those of continuously sufficient animals, while imposing deficiency after an initially adequate exposure impaired performance to a similar extent as chronic deficiency [126]. Pharmacological inhibition of cyclooxygenase (COX) enzyme also improved spatial recognition in deficient mice, suggesting that altered ARA–COX-derived signaling may contribute to the cognitive phenotype, although this pathway was not established as the sole mechanism [126]. Together, these findings indicate that cognitive outcomes remain responsive to subsequent nutritional environments and that altered ARA–COX signaling may contribute to the functional consequences of *n*-3 PUFA deficiency.

This distinction may help reconcile the less consistent cognitive effects observed in human supplementation trials. Human studies predominantly evaluate DHA or DHA-containing *n*-3 PUFA supplementation in populations not selected for demonstrated n-3 deficiency and with heterogeneous or incompletely characterized baseline status; accordingly, broad cognitive benefits are generally small, inconsistent, or absent despite biological changes in FA exposure [116,117,118,130]. By contrast, experimental models more often examine marked deficiency or defined developmental insults, conditions in which the biological requirement for *n*-3 PUFAs becomes easier to detect. As summarized in Table 2, animal studies encompass deficiency, repletion, and developmental challenge paradigms, whereas human studies predominantly assess supplementation or observational exposure. This distinction helps explain why null supplementation effects do not necessarily imply absence of biological function, but rather a context-dependent response shaped by nutritional status, developmental timing, and the cognitive domain assessed [119,120,129].

## 6. Limitations

As a structured narrative review, this review was designed to integrate heterogeneous mechanistic, experimental, observational, and randomized evidence rather than to provide a systematic quantitative synthesis or pooled effect estimate. Although the literature search was structured and updated through July 2026, study identification and weighting remain less standardized than in a systematic review, and no formal risk-of-bias or certainty-of-evidence assessment was performed. The evidence base itself is also highly heterogeneous. Human studies differ substantially in baseline dietary *n-*3 PUFA intake and fatty-acid status, supplement composition and dose, timing and duration of exposure, adherence, comparator diets, age at follow-up, and neurodevelopmental instruments, while many trials did not specifically enroll participants with demonstrated *n*-3 PUFA deficiency. Observational studies are additionally susceptible to residual confounding by maternal diet, education, socioeconomic status, supplement use, and postnatal environmental factors. Experimental models provide greater control over exposure and developmental timing but often rely on marked *n*-3 PUFA deficiency, controlled dietary formulations, or defined developmental insults that do not fully reproduce the more heterogeneous nutritional conditions observed in humans. Differences in species-specific brain development, lipid metabolism, exposure windows, and behavioral outcomes further limit direct translation. These methodological and biological differences should therefore be considered when interpreting discrepancies between experimental and human findings and when identifying the populations and developmental windows most likely to benefit from *n-*3 PUFA interventions.

## 7. Conclusions

*n-*3 PUFAs, particularly DHA, are essential for normal fetal and infant brain development by supporting membrane organization, synaptic maturation, lipid signaling, and neural plasticity. Maternal metabolism, placental transfer, and postnatal supply through breast milk determine DHA availability during critical developmental windows. Ensuring an adequate maternal intake of *n*-3 PUFAs is essential for preventing neurodevelopmental disorders and protecting brain maturation, recognizing that the effects of nutritional interventions depend on the mother’s prior nutritional status, critical windows of exposure, and environmental demands. However, the biological requirement for DHA should be distinguished from the effects of supplementation above adequacy, as human trials have not consistently shown improvements in global cognitive outcomes. Overall, current evidence supports a context-dependent model in which the effects of *n*-3 PUFA availability vary according to nutritional status, developmental timing, biological challenge, and the cognitive domain assessed. Future studies should identify the populations and developmental windows in which *n*-3 PUFA interventions are most likely to provide measurable neurodevelopmental benefit.

## Figures and Tables

**Figure 1:**
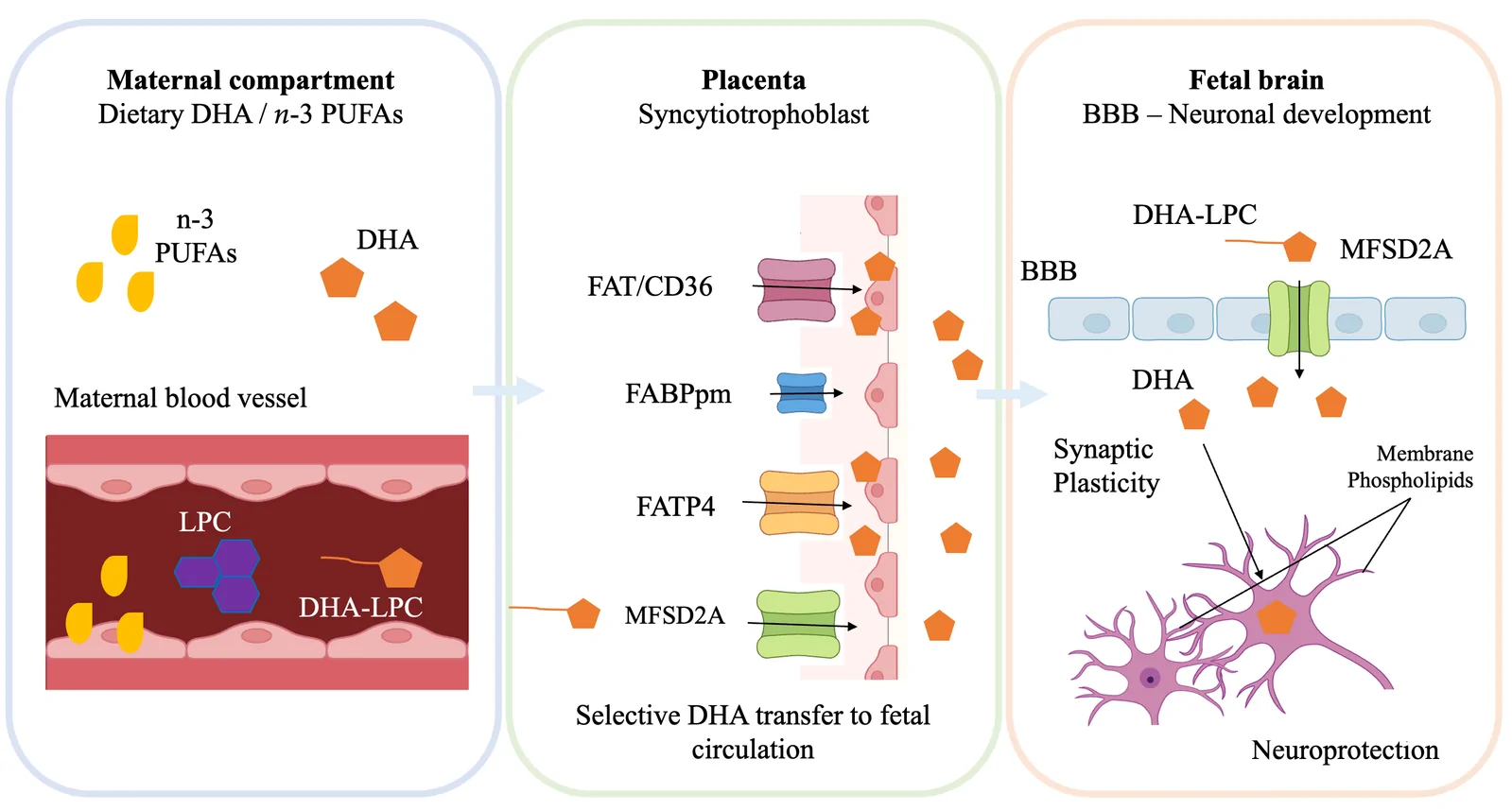
Maternal–placental–brain axis linking *n*-3 PUFA availability to DHA transport during neurodevelopment. Maternal DHA availability contributes to selective maternal–fetal fatty acid transfer, followed by LPC-DHA uptake across the blood–brain barrier through MFSD2A and incorporation into the developing brain. Abbreviations: BBB, blood–brain barrier; DHA, docosahexaenoic acid; FABPpm, plasma membrane fatty acid-binding protein; FAT/CD36, fatty acid translocase/CD36; FATP4, fatty acid transport protein 4; LPC, lysophosphatidylcholine; MFSD2A, major facilitator superfamily domain-containing protein 2A; PUFAs, polyunsaturated fatty acids [20,21,22]. Original work.

**Figure 2:**
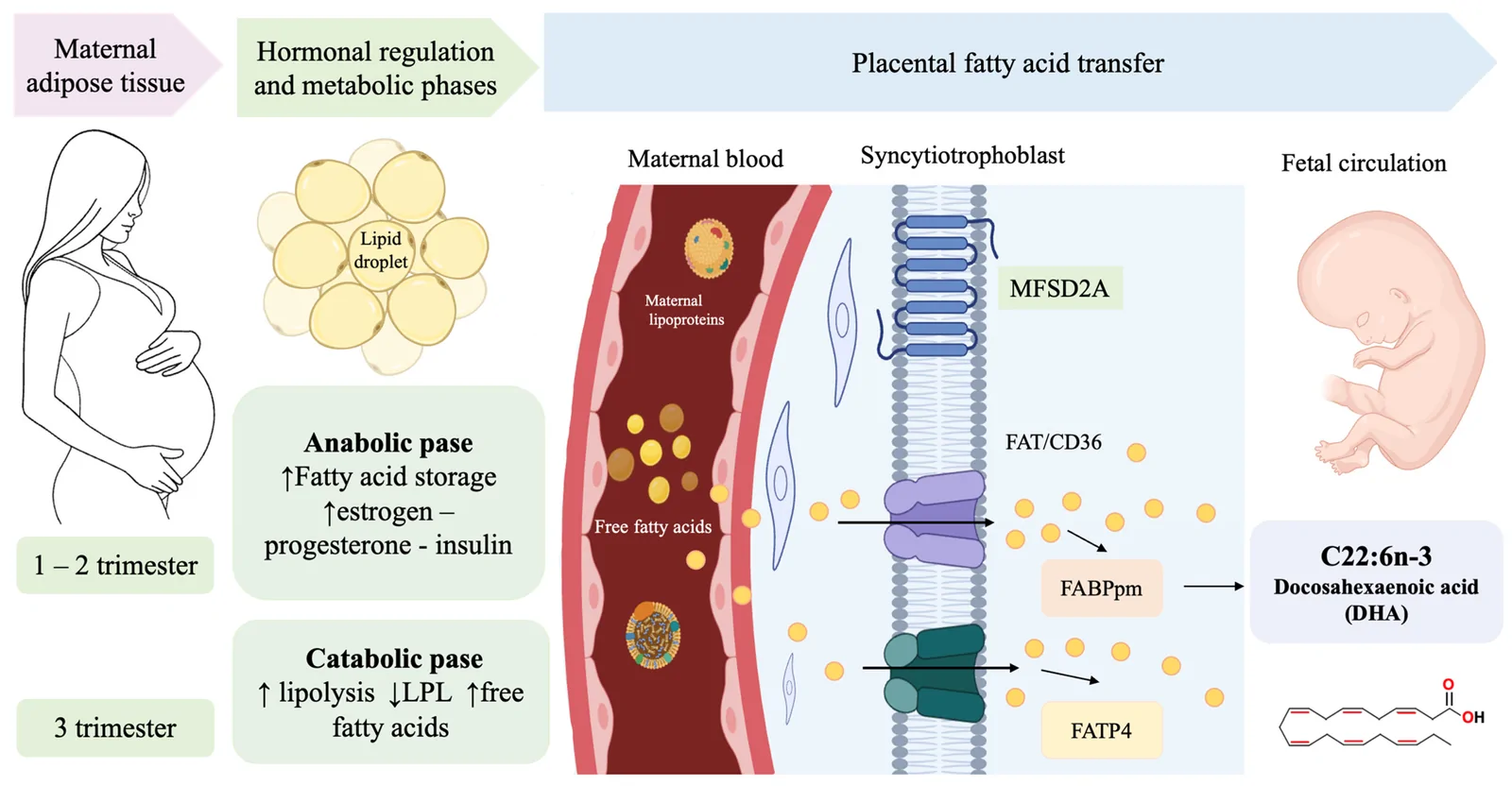
Maternal lipid metabolism and placental DHA supply during pregnancy. Maternal metabolic adaptations shift from lipid storage during the anabolic phase to increased lipolysis and increased availability of circulating fatty acids during late gestation, supporting placental transfer to the fetal circulation. Abbreviations: MFSD2A, major facilitator superfamily domain-containing protein 2A; DHA, docosahexaenoic acid; FABPpm, plasma membrane fatty acid-binding protein; FAT/CD36, fatty acid translocase/CD36; FATP4, fatty acid transport protein 4; LPL, lipoprotein lipase [27,28,29]. Original work.

**Figure 3:**
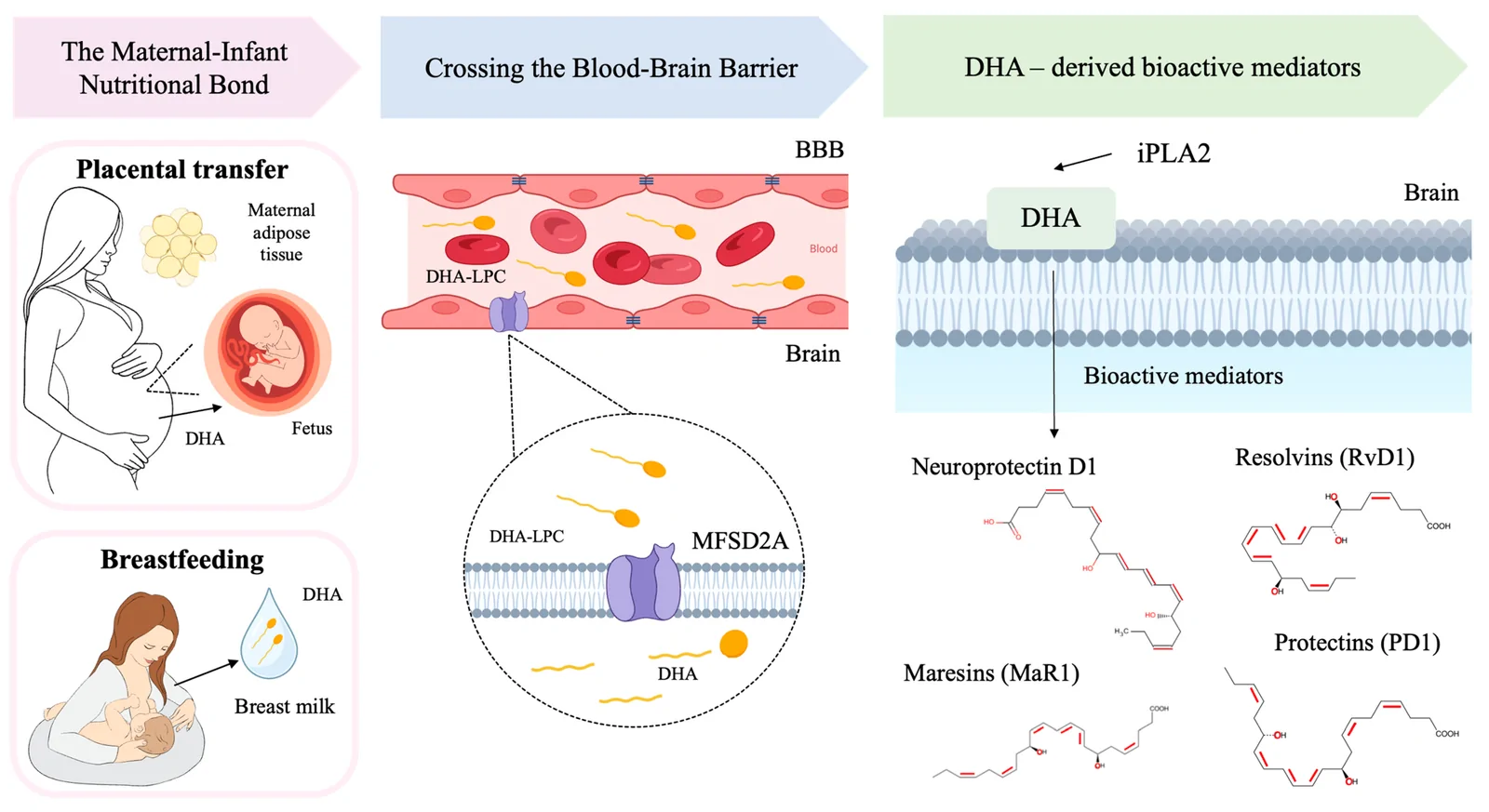
Maternal DHA supply, brain uptake, and DHA-derived bioactive mediators during neurodevelopment. Maternal DHA is supplied to the offspring through placental transfer and breastfeeding, while LPC-DHA is transported across the blood–brain barrier through MFSD2A. Following incorporation into brain membrane phospholipids, DHA can be released by calcium-independent phospholipase A_2_ and enzymatically converted into specialized bioactive mediators. Abbreviations: BBB, blood–brain barrier; DHA, docosahexaenoic acid; iPLA_2_, calcium-independent phospholipase A_2_; LPC, lysophosphatidylcholine; MFSD2A, major facilitator superfamily domain-containing protein 2A; MaR1, maresin 1; PD1, protectin D1; RvD1, resolvin D1 [21,70]. Original work.

**Table 1:** Comparison of n-3 PUFA transport in normal pregnancy and in cases of obesity.

*n*-3 PUFAs	Normal Pregnancy	Obesity Pregnancy
Protein transport at the placental level	Efficient coordination: FAT/CD36, FABPpm, FATP4, and MFSD2A for the hydrolysis and uptake of lipids [27,29].	Altered protein expression: compensatory ↑ MFSD2A in the placenta [73].
DHA accumulation	Placental biomagnification: preferential transfer of DHA, resulting in fetal concentrations ↑ than maternal concentrations [36].	A ↓ in the metabolic accumulation rate and ↓ ability of placental tissue to incorporate and retain DHA [74].
Lipid composition in the placenta	Selective enrichment with PUFAs (DHA and ARA) necessary for embryonic development [70].	Larger placentas but with ↓ levels of DHA and ARA, and ↑ SFAs such as PA [37,38].
Transport at the brain level	Active uptake of LPC-DHA mediated by the MFSD2A transporter in the fetal cerebral microvasculature [21].	Compensatory ↑ MFSD2A expression in the brain, which does not result in ↑ DHA transport, suggesting a protective mechanism [75].

**Abbreviations:** PUFAs: polyunsaturated fatty acids, *n*-3: series *n*-3, FAT/CD36: fatty acid translocase/CD36, FABPpm: plasma membrane fatty acid-binding protein, FATP4: fatty acid transport protein 4, LPC: lysophospholipids, MFSD2A: transport proteins and the major facilitator superfamily domain-containing protein 2a, SFAs: saturated fatty acids, PA: palmitic acid, DHA: docosahexaenoic acid (C22:6 *n*-3), ARA: arachidonic acid (C20.4*n*-6). ↑, increased/higher; ↓, decreased/lower/reduction.

## Data Availability

No new data were created or analyzed in this study. Data sharing is not applicable to this article.

## Abbreviations

ALA	alpha-linolenic acid (C18:3*n*-3)
ARA	arachidonic acid (C20:4*n*-6)
SFAs	saturated fatty acids
BBB	blood–brain barrier
BDNF	brain-derived neurotrophic factor
COX	cyclooxygenase enzyme
CREB	cAMP response element-binding protein
DHA	docosahexaenoic acid (C22:6*n*-3)
EPA	eicosapentaenoic acid (C20:5*n*-3)
FABPpm	plasma membrane fatty acid-binding protein
FAT/CD36	fatty acid translocase/CD36
FATP	fatty acid transport protein
FATP4	fatty acid transport protein 4
HPA	hypothalamic–pituitary–adrenal
LA	linoleic acid (C18:2*n*-6)
LC-PUFAs	long-chain polyunsaturated fatty acids
LOX	lipoxygenase
LPC	lysophosphatidylcholine
LPL	lipoprotein lipase
LTP	long-term potentiation
MFSD2A	major facilitator superfamily domain-containing protein 2A
NF-κB	nuclear factor kappa B
NLRP3	NLR family pyrin domain-containing 3
PA	palmitic acid (C16:0)
PKC	protein kinase C
PUFAs	polyunsaturated fatty acids
